# The quality of group interactions in medical problem–based learning in China: the roles of intercultural sensitivity and group ethnic composition

**DOI:** 10.1186/s12909-023-04616-3

**Published:** 2023-09-13

**Authors:** Rong Wang, Chuanyong Liu, Shu-Yong Zhang

**Affiliations:** 1 Department of Physiology and Pathophysiology, School of Basic Medical Sciences, Shandong University, Jinan, 250012 China; 2 School of Chemistry and Chemical Engineering, Shandong University, Jinan, 250100 China

**Keywords:** PBL, Interaction, Collaborative learning, Intercultural sensitivity, Foreign students, Mixed group

## Abstract

**Background:**

Chinese universities are increasingly recruiting foreign students, and problem-based learning (PBL) is an effective approach to integrating those students. This study focuses on the role of intercultural sensitivity and group ethnic composition on the quality of group interaction in medical problem-based learning in China.

**Methods:**

This paper reports an investigation of the differences in three types of group interaction (exploratory questions, cumulative reasoning, and handling conflict) among 139 s-year medical undergraduates from two backgrounds (Chinese and foreign) in a PBL setting. The roles of intercultural sensitivity, group ethnic composition, and students’ personal characteristics including age, gender and ethnicity on students’ perceptions of the three types of interaction were quantitatively analyzed. A 35-item questionnaire and demographic survey were administered to second year medical undergraduates.

**Results:**

The results indicated that group ethnic composition was a significant negative predictor while intercultural sensitivity was a strong positive predictor of group interactions involving exploratory questions and cumulative reasoning. In addition, group heterogeneity in terms of age and ethnicity were significant predictors of group interaction.

**Conclusions:**

The findings of this study provide insights for strategically designing effective multiethnic group learning environments that encourage interaction and collaboration.

## Introduction

With the globalization of medical education, educational institutions, including those in some eastern countries such as China, Japan, and South Korea, are increasingly recruiting foreign students [1, 2]. It is widely accepted that the extensive integration of international and domestic students is necessary to fully benefit from internationalization [3, 4]. Although many educational institutions have developed learning models such as small group learning, learning communities, and campus schools to integrate international students [4–6], higher education researchers continue to explore ways to optimize positive group learning outcomes in cross-cultural learning environments [7]. This is a relatively new phenomenon in medical education, and studies have suggested that many universities still face challenges related to cross-cultural environments and curricular adaptations [6, 8–10].

Medical problem-based learning (PBL), a form of small group learning, involves the integration of basic and clinical skills, and collaborative learning is a central element [11, 12]. Interactions in PBL [13] have been found to be positively related to the success of tutorial groups [14, 15]. Group interactions may be a key predictor of successful collaborative learning. Since the early 1990s, an increasing number of studies have focused on how group ethnic composition affects the quality of collaborative learning [16, 17]. These studies have revealed both advantages and disadvantages. Wright and Lander [18] found that the verbal participation of Southeast Asian students was inhibited when they were in a group with Australian students, suggesting that “there can be little assurance that arranging groups of mixed-ethnic membership will lead to profitable intercultural interaction” (p. 250). The possible influencing factors included the lack of language proficiency, familiarity with the cultural environment, and experience in collaborative groups [18]. However, many studies have found that mixed ethnic groups enhance student interactions across diverse boundaries and that students in such groups receive better performance evaluations than students in homogenous groups [4, 5, 16]. What remains unknown is how multiethnic groups affect student learning outcomes or group interactions in a PBL learning model. What other factors influence the quality of student interaction in multiethnic small group learning?

Most studies have examined group interactions using observational data as an instrument, which could be difficult to manage [19, 20]. Moreover, those studies have mainly focused on two aspects of student interactions: asking and answering questions and reasoning. Van Boxtel [21] emphasized that three interaction dimensions are essential for stimulating deep learning: asking and answering questions, reasoning, and resolving conflict (or exploratory questions, cumulative reasoning, and handling conflicts). Pleijers et al. [22] developed a corresponding questionnaire to effectively assess the quality of group interactions in PBL. There are still gaps in the literature around the empirical studies about the impact of ethnic composition on the three interaction dimensions in PBL. Additionally, a general weakness of most studies on the effect of group ethnic composition on their interactions is that only a few personal-level variables related to culture have been analyzed [16, 18, 23, 24]. However, other individual variables related to culture may explain the differences between the interactions of ethnically homogenous and mixed groups. We use the Sociocultural Theory [25–27] as our conceptual basis to identify factors that could contribute to interactions in groups. The Sociocultural Theory posits that culture make an impact on students’ learning behavior (e.g., group interactions) [28]. According to the sociocultural perspective of collaborative learning [29, 30], cultural diversity affects collaborative learning by influencing behavioral process (group interactions). Applied these perspectives to the medical PBL, a student is more likely to succeed when he or she able to better understand and appreciate cultural differences and maintain a positive emotion. Intercultural sensitivity is an interesting variable in this context. As the most essential element of cultural competence, intercultural sensitivity is defined as “an individual’s ability to develop a positive emotion towards understanding and appreciating cultural differences that promotes appropriate and effective behavior in intercultural communication” [31]. Cultural competence is indeed considered as one of the core competences in medical professionalism, and it implies that an individual has the ability to think in a multicultural environment and to make their behavior show respect for other cultures [32]. Intercultural sensitivity may be regarded as one of the dimensions of intercultural competence, which involves a gradual change in emotions and awareness that help with developing skills for interacting with people of diverse backgrounds [33]. Studies have identified various tools to assess intercultural sensitivity. A widely cited approach is that of Chen and Starosta [34], who developed an intercultural sensitivity scale (ISS). There is general agreement that intercultural sensitivity is a prerequisite for competent cross-cultural communication [31, 35]. Based on the Sociocultural Theory, sociocultural perspective of collaborative learning and the properties of intercultural sensitivity, it is probable that intercultural sensitivity could play crucial moderating roles in PBL by affecting interaction processes in group. As cultural diversity is closely related to behavioral process in culturally heterogeneous groups [36], intercultural sensitivity is likely to moderate the effects of cultural diversity on the group interactions. However, little empirical research has been conducted on how intercultural sensitivity influences the quality of group interactions in cross-cultural PBL.

To contribute to the literature on the factors that influence group interactions, this study investigates the influence of group ethnic composition and the relationship between students’ intercultural sensitivity and their group interactions in medical PBL.

## Methods

### Curricula background

The PBL approach can be summarized as including the stages of identifying and deconstructing problems, thinking and choosing action strategies based on knowledge and experience, and then critically evaluating and reflecting on the actions [37]. In medical PBL curricula in Shandong University, second year medical students are assigned to learning groups (9–10 students per group) according to their academic performance. The PBL curriculum was comprised of 12 weekly class meetings of 100 min each. The content of each case containing an authentic problem need to be solved is presented in the first introductory session. Students in each group identify their learning needs related to the problem and derive the learning objectives, and discuss in the second and third discussion sections (a few days after conducting research on the assigned learning objectives). The basic theme of the course is related to anatomy, physiology, pathophysiology, pharmacology or biochemistry.

All PBL tutors were given standardized training in the weekly pre-PBL session tutors’ meetings about the basic rules for introduction of cases [38]. Tutors were required to use a guided questioning approach. Guiding questions (e.g., “What is the pathophysiological mechanism?”) were asked by the tutor during the student discussion. At the end of the semester, the students were required to give feedback on the contents of PBL case and PBL methodology, the peers and tutors’ performance, and any other suggestions they wished to make about the course.

### Participants

Based on their prior academic performance, 139 medical students were assigned to 15 groups of 9–10 participants (high, average and low achievement level) before the start of the curriculum. The proportions of high-achieving, average, and low-achieving students in each group were almost equal. The participants were Year 2 students at the Basic School of Medicine, Shandong University. Eighty-six participants were Chinese (ethnicity coded 1), and fifty-three international students (ethnicity coded 2) from six foreign countries such as India, Pakistan, and Syria. Sixty-five female participants comprised of the sample, and seventy-four male participants. Among the Chinese students, fifty-one participated in a Chinese-language discussion of PBL, and thirty-five participated in an English-language discussion of PBL. All of the foreign students participated in an English-language discussion of PBL. Eight of the 15 groups (*n* = 72) were either all Chinese students (*n* = 51) or all foreign students (*n* = 21). These groups were labelled unmixed group (coded 1). The remaining seven groups (*n* = 67) included both Chinese and foreign students and were labelled mixed groups (coded 2). The mixed groups contained almost equal numbers of Chinese and foreign students. The study data were collected using a personal characteristics form (four questions about age, gender, ethnicity, ethnic group), the ISS, and a questionnaire about the quality of group interaction in PBL. All of the participants completed the questionnaire, and the final sample size was 139. The study was approved by the Research Ethics Committee of the Basic School of Medicine, Shandong University.

### Measures

At the end of the semester, the students completed the following 35-items questionnaire regarding their intercultural sensitivity and quality of group interactions in PBL (Table 1).

**Table 1:** Factor analysis of the questionnaire used in this study

**The KMO and Bartlett’s test in the section**	**Variable (number of items)**	**Item**	**Factor**							
			1	2	3	4	5	6	7	8
**Intercultural Communication Sensitivity** (KMO = 0.76, Bartlett test of sphericity: χ^2^(276) = 2196.46, *p* < 0.00, total variance explained = 72.14%)	Interaction engagement (7)	I enjoy interacting with people from different cultures	.67							
		I tend to wait before forming an impression of culturally-distinct counterparts	.79							
		I am open-minded to people from different cultures	.73							
		I often give positive responses to my culturally different counterpart during our interaction.	.65							
		I avoid those situations where I will have to deal with culturally-distinct persons.	.63							
		I often show my culturally-distinct counterpart my understanding through verbal or nonverbal cues.	.68							
		I have a feeling of enjoyment towards differences between my culturally-distinct counterpart and me	.58							
	Respect for cultural differences (6)	I think people from other cultures are narrow-minded		.56						
		I don’t like to be with people from different cultures.		.72						
		I respect the values of people from different cultures		.81						
		I respect the ways people from different cultures behave		.78						
		I would not accept the opinions of people from different cultures		.77						
		I think my culture is better than other cultures.		.65						
	Interaction confidence (5)	I am pretty sure of myself in interacting with people from different cultures			.74					
		I find it very hard to talk in front of people from different cultures			.74					
		I always know what to say when interacting with people from different cultures			.83					
		I can be as sociable as I want to be when interacting with people from different cultures			.78					
		I feel confident when interacting with people from different cultures			.79					
	Interaction enjoyment (3)	I get upset easily when interacting with people from different cultures				.76				
		I often get discouraged when I am with people from different cultures				.85				
		I often feel useless when interacting with people from different cultures				.55				
	Interaction attentiveness (3)	I am very observant when interacting with people from different cultures					.60			
		I try to obtain as much information as I can when interacting with people from different cultures					.57			
		I am sensitive to my culturally-distinct counterpart’s subtle meanings during our interaction					.72			
**Quality of group interactions** (KMO = 0.84, Bartlett test of sphericity: χ^2^(55) = 919.80, *p* < 0.00, total variance explained = 66.05%)	Exploratory questions (4)	Students posed adequate questions to each other in order to understand the learning content						.75		
		What group members said was checked by asking each other critical questions						.76		
		A group member who was formulating an explanation concerning the problem asked in between times whether his/her explanation was right						.74		
		One explanation did not suffice for the group members; alternative explanations were also mentioned						.83		
	Cumulative reasoning (4)	Group members elaborated on each other’s arguments							.76	
		When someone argued something, then that statement was motivated							.69	
		Explanations of group members were completed with explanations of other group members							.74	
		Students drew conclusions from the information that was discussed in the group							.84	
	Handling conflict (3)	In the group, some contradictory beliefs on information concerning the learning content were present								.78
		One or more group members was/were contradicted by the others								.91
		When someone contradicted a group member, that person stated a counter-argument								.83

#### Assessment of students’ intercultural sensitivity

Intercultural sensitivity was measured using items adapted from the ISS (α = 0.76) developed by Chen and Starosta [34]. Chinese students use Mandarin version of ISS, and foreign students use English version of ISS. Despite the fact that ISS has conducted research in the Chinese context, we have not been able to obtain the Chinese version of ISS from previous publications, so we decided to translate the scale into Chinese as suggested by Van de Vijver et al. [39] for the translation of scales in cross-cultural studies. The first author was responsible for translation from the English text to Chinese, and then the second author was responsible for translation from the Chinese version back to English again. The research assistant compares the two versions in different languages. When there are significant differences between the two versions, the research assistant will discuss them with the two translators until the three parties agree on a final translation. The 24 items (see Table 1) were rated on a 5-point Likert scale (1 = strongly disagree to 5 = strongly agree) and assessed five dimensions: interaction engagement (seven items, e.g., “I tend to wait before forming an impression of culturally distinct counterparts”), respect for cultural differences (six items, e.g., “I respect the ways people from different cultures behave”), interaction confidence (five items, e.g., “I feel confident when interacting with people from different cultures”), interaction enjoyment (three items, e.g., “I often get discouraged when I am with people from different cultures”), and interaction attentiveness (three items, e.g., “I try to obtain as much information as I can when interacting with people from different cultures”). Reverse-scored items were recoded to ensure that higher scores always indicated greater intercultural sensitivity. The ISS was valid in its integrated form [34], and the aggregate Cronbach’s Alpha was 0.85.

#### Assessment of the quality of group interactions

The quality of group interactions in PBL was measured using an 11-item questionnaire (see Table 1) based on scales adapted from Pleijers et al. [22], and it showed good reliability (α = 0.90). The scale assessed three types of group interactions: exploratory questions, cumulative reasoning, and handling conflict. The items were rated on a 5-point Likert scale (1 = *strongly disagree* to 5 = *strongly agree*).

## Analyses

IBM SPSS 23.0 was used to examine the reliability of the subscales used in this study as assessed by Cronbach’s α coefficients. Exploratory factor analysis (EFA) was used to determine the factor structure. Descriptive statistics and correlations were computed for all of the variables. One-way ANOVA tests were performed to analyze the students’ ethnicity and group ethnic composition in relation to intercultural sensitivity and group interactions in the PBL setting. Then hierarchical multiple regression was conducted to examine the statistical predictive power of ethnic composition, intercultural sensitivity, and personal characteristics (age, gender, and ethnicity). The participants’ age, gender, and ethnicity were entered in model 1. In model 2, ethnic composition was entered, and intercultural sensitivity was entered in model 3.

## Results

### Reliability and exploratory factor analysis (EFA) for validity

The Cronbach’s α for all variables ranged from 0.83 to 0.85, which indicated that all constructs in this study had a satisfactory internal consistency (Table 2). The exploratory factor analysis was performed on twenty-four items (ISS questionnaire) and eleven items (The quality of group interactions in PBL questionnaire) with Varimax rotation conducting the principal component analysis. To assess the appropriateness of the sampling for factor analysis, the Kaiser– Meyer–Olkin (KMO) measure and Bartlett test of sphericity were used. The results show that KMO values for the two questionnaires were 0.76 and 0.84, above the recommended value of 0.60 (Table 1). Bartlett’s test of sphericity for two questionnaires was also significant (χ2 (276) = 2196.46, *p* < 0.00 and χ2 (55) = 919.80, *p* < 0.00). Given these results, factor analysis was suitable with all of the 35 items. Varimax rotation of the factor loading matrix was used to examine the solutions for the eight factors namely Interaction engagement (Factor 1), Respect for cultural differences (Factor 2), Interaction confidence (Factor 3), Interaction enjoyment (Factor 4), Interaction attentiveness (Factor 5), Exploratory questions (Factor 6), Cumulative reasoning (Factor 7) and Handling conflict (Factor 8). The factor loading matrix was shown in Table 1. The criteria for selection was to remove items with factor loads below 0.40. The factor loadings of 24 items (ISS questionnaire) and 11 items (The quality of group interactions in PBL) in this study were considered to be sufficient to explain more than 65% of the variance, respectively.

**Table 2:** Descriptive statistics, distributional properties and correlation coefficients for all of the variables

No.	Variables	1	2	3	4	5	6	7	8
1	Mean age	-							
2	Gender	-0.16	-						
3	Ethnicity	0.72**	-0.14	-					
4	Ethnic group composition	0.22**	-0.07	0.19*	-				
5	Intercultural Communication Sensitivity	0.16	0.15	0.39**	0.13	-			
6	Exploratory questions	0.16	0.09	0.08	-0.19*	0.42**	-		
7	Cumulative reasoning	0.06	0.21	0.04	-0.20*	0.46**	0.79**	-	
8	Handling conflicts	0.03	-0.04	-0.18*	-0.1	0.01	0.34**	0.36**	-
	Cronbach’s alpha (α)	-	-	-	-	0.85	0.85	0.84	0.83
	Mean	21.46	0.47	1.38	1.48	3.69	3.92	3.96	3.74
	SD (*n* = 139)	1.91	0.5	0.49	0.50	0.39	0.65	0.69	0.76
	Skewness	1.29	0.13	0.49	0.07	-0.08	-0.49	-0.50	-0.71
	Kurtosis	2.95	-2.01	-1.78	-2.02	-0.47	0.87	0.71	1.18

^*^p < .05, **p < .01

### Descriptive statistics and correlations

The descriptive statistics (mean, SD), distributional properties (skewness, kurtosis), and correlation coefficients for all of the variables are presented in Table 2. As age, gender, and ethnicity also affect group dynamics [40–43], they were used as additional independent variables. The mean age of the participants was 21.46 years (SD = 1.91). Female participants comprised 46.8% (*n* = 65) of the sample, and male participants 53.2% (*n* = 74). Chinses participants were 86 (61.9%), and international students were 53 (38.1%) from six foreign countries such as India, Pakistan, and Syria. According to Curran, West, and Finch [44], skewness values less than 2 and kurtosis values less than 7 can be accepted as the critical values of a normal distribution. The Pearson correlation coefficients showed that intercultural sensitivity was positively correlated with group interactions related to exploratory questions and cumulative reasoning, whereas group ethnic composition was negatively related to those two types of group interactions. The participants’ ethnicity was significantly correlated with group interactions related to handling conflict (Table 2).

### Intercultural sensitivity and group interactions in ethnically unmixed and mixed groups

Table 3 shows the distribution of the mean intercultural sensitivity scores and three types of group interaction in PBL according to their characteristics. One-way ANOVA tests were conducted to analyze ethnicity and group ethnic composition in relation to intercultural sensitivity and the three types of group interaction: exploratory questions, cumulative reasoning, and handling conflict (see Table 3). The foreign students had higher intercultural sensitivity scores than the Chinese students (*p* < 0.001) but lower scores for interactions related to handling conflict (*p* < 0.05) (Table 3). The students in the ethnically unmixed groups had significantly less group interaction related to exploratory questions and cumulative reasoning (*p* < 0.05). To examine the effects of group ethnic composition on the Chinese and foreign students, one-way ANOVA tests were conducted separately for each set of students to identify differences related to intercultural sensitivity and the three types of group interaction (see Table 3). For the Chinese students, group ethnic composition was a significant factor that decreased interactions related to exploratory questions (*F* [1,84] = 16.66, *p* < 0.001) and cumulative reasoning (*F* [1, 84] = 11.39, *p* < 0.001). However, group ethnic composition did not affect the Chinese students’ intercultural sensitivity. For the foreign students, ethnic composition had no significant impact on intercultural sensitivity or the three types of group interaction.

**Table 3:** Distribution of scale mean scores according to characteristics of the medical students (*n* = 139)

Characteristics	Intercultural Sensitivity		Exploratory questions		Cumulative reasoning		Handling conflicts	
	X ± SD	Test and *p*	X ± SD	Test and *p*	X ± SD	Test and *p*	X ± SD	Test and *p*
Ethnicity								
Chinese students (*n* = 86)	3.57 ± 0.38	*F*:25.15	3.89 ± 0.57	*F*:0.77	3.94 ± 0.62	*F*:0.17	3.85 ± 0.63	*F*:4.45
Foreign students (*n* = 53)	3.88 ± 0.33	***p*:0.000**	3.99 ± 0.75	*p*:0.38	4.00 ± 0.80	*p*:0.68	3.57 ± 0.92	***p*:0.04**
Ethnic group composition								
Unmixed groups (*n* = 72)	3.68 ± 0.38	*F*:0.11	4.05 ± 0.55	*F*:5.38	4.10 ± 0.58	*F*:5.69	3.82 ± 0.65	*F*:1.50
Mixed groups (*n* = 67)	3.70 ± 0.40	*p*:0.74	3.79 ± 0.71	***p*:0.02**	3.82 ± 0.78	***p*:0.02**	3.66 ± 0.86	*p*:0.22
Ethnicity*Ethnic group composition								
Chinese students in Unmixed groups (*n* = 51)	3.59 ± 0.36	*F*:0.21	4.08 ± 0.47	*F*:16.66	4.12 ± 0.50	*F*:11.39	3.88 ± 0.57	*F*:0.23
Chinese students in Mixed groups (*n* = 35)	3.55 ± 0.40	*p*:0.65	3.62 ± 0.59	***p*:0.000**	3.69 ± 0.70	***p*:0.001**	3.81 ± 0.70	*p*:0.63
Foreign students in Unimixed groups (*n* = 21)	3.90 ± 0.32	*F*:0.16	3.96 ± 0.72	*F*:0.03	4.04 ± 0.74	*F*:0.09	3.68 ± 0.80	*F*:0.50
Foreign students in Mixed groups (*n* = 32)	3.87 ± 0.34	*p*:0.69	4.00 ± 0.78	*p*:0.87	3.97 ± 0.85	*p*:0.77	3.50 ± 0.99	*p*:0.48

### The impact of group ethnic composition and intercultural sensitivity on group interactions

To analyze how the variables predicted the three types of group interaction, multiple hierarchical regression analyses were conducted for each type of group interaction (exploratory questions, cumulative reasoning, and handling conflict). The individual characteristic variables for the groups (gender, mean age, and ethnicity) were entered in model 1. In model 2, group ethnic composition was added. Finally, the ISS scores were added in the model 3. The regression results are shown in Table 4. Group ethnic composition negatively predicted interactions that involved exploratory questions and cumulative reasoning, whereas intercultural sensitivity significantly and positively predicted those types of group interactions. The addition of group ethnic composition in model 2 strengthened the correlation with the two types of group interactions (*p* < 0.01). The addition of the ISS scores in model 3 further significantly increased the explained variance (*p* < 0.001). Neither intercultural sensitivity nor group ethnic composition were correlated with interaction involving handling conflict. The effects of age and ethnicity on the three types of interaction were significant, but gender did not explain a significant amount of the variance in the three types of group interaction in PBL, as shown in Table 4.

**Table 4:** Summary of hierarchical regression analysis predicting group interactions (*n* = 139)

Variable	Exploratory questions				Cumulative reasoning				Handling conflicts			
	β	*R*^2^	Δ*R*^2^	ΔF	β	*R*^2^	Δ*R*^2^	ΔF	β	*R*^2^	Δ*R*^2^	ΔF
**Model 1**		0.04	0.04	1.97		0.05	0.05	2.52		0.09^**^	0.09	4.2^**^
Mean age	0.23				0.1				0.32^*^			
Gender	0.12				0.23				-0.05			
Ethnicity	-0.07				-0.002				-0.42**			
**Model 2**		0.09^**^	0.05	7.76^**^		0.1^*^	0.05	6.61^*^		0.1	0.01	1.53
Mean age	0.27^*^				0.13				0.34**			
Gender	0.11				0.22^*^				-0.06			
Ethnicity	-0.06				0.01				-0.41**			
Ethnic group composition	-0.24**				-0.22*				-0.1			
**Model 3**		0.29^***^	0.2	36.94^***^		0.31^***^	0.21	40.95^***^		0.12	0.02	3.16
Mean age	0.38**				0.25^*^				0.38^**^			
Gender	0.02				0.12				-0.09			
Ethnicity	-0.36**				-0.3*				-0.51***			
Ethnic group composition	-0.23**				-0.21**				-0.1			
Intercultural Sensitivity	0.5^***^				0.52^***^				0.16			

^*^p < .05, * *p < .01, ***p < .001

## Discussion

This study provides a novel examination of group ethnic composition and intercultural sensitivity as predictors of group interactions in PBL among medical students. We found that intercultural sensitivity, group ethnic composition, age, and ethnicity are significant predictors of group interactions in PBL among medical students. In this study, the average ISS score of the Chinese students was lower than that of the international students. To the best of our knowledge, this study is the first to report on the intercultural sensitivity among Chinese and international medical undergraduates in China’s mainland.

The first research question was whether group interactions in PBL vary between ethnically mixed and unmixed groups. We found that ethnically mixed and unmixed groups do differ in their interaction types. Specifically, mixed groups engage in significantly fewer interactions that involve exploratory questions and cumulative reasoning than unmixed groups. Regarding the effect of a group’s ethnic composition on students of different ethnicities, the difference in the types of interactions in ethnically unmixed versus mixed groups was significant for the Chinese students but not for the international students. These results are consistent with the literature from several countries [18, 45, 46], suggesting that interaction in learning process between domestic and international students remains limited. Moreover, our study also partially echoes Hope et al.’s study [47] that assessed team development of student across ethnicity through Tuckman’s four team-development stages (Forming, Storming, Norming, Performing). One of the study cohorts stuck in the third stage- Norming and unable to move forward to reach the last stage- Performing. Performing stage is characterized by action-filled and active interactions between participants [48]. According to Hope et al.’s suggestion, possible factors explaining why mixed groups in our study do not reach the ideal performing stage are inconsistent learning goals, failure to form subgroups to clarify their respective learning goals, and the learning limited to the classroom. Therefore, the quality of interaction in mixed group may be facilitated to reach the performing stage by dividing into multiple subgroups each with a distinct goal and receiving good community support, etc. The Chinese medical students’ lower ISS scores relative to those of the international students in this study, which is consistent with the intercultural sensitivity level report of Chinese students majoring in science and technology [49], may also at least partially explain this result. Foreign study experience is generally believed to significantly improve intercultural sensitivity [50, 51], so it is not surprising that the foreign students had higher ISS scores than the Chinese students, as they had had at least one year of study experience abroad (in China). Most of the Chinese students had no such experience. According to the definition of intercultural sensitivity, student with higher ISS level could better develop a positive emotion towards cultural diversity and thus promotes effective behavior in intercultural interactions. The lower level of ISS among Chinese students compared to international students may be responsible for their inhibited interaction [31]. Another potential explanation for the significant difference in the two types of interaction by Chinese students in ethnically mixed groups could be a lack of language proficiency. In the unmixed groups of Chinese students, they interacted in Chinese; however, in the mixed groups, only English, a non-native language, could be used for interactive communication. As the foreign students spoke English in both the unmixed and mixed groups, their group interactions did not vary between the two groups. The Chinese students’ participation may have been reduced because they lacked confidence in their language skills and feared losing face because of it [52]. In addition to the lack of confidence associated with a lack of language skills, the reduced level of interaction by Chinese students in the ethnically mixed groups may be explained by Byrne’s similarity–attraction theory [53], which proposes that individuals prefer to build relationships with those who are similar to them [54, 55]. However, that does not explain the lack of a difference between the foreign students’ interactions in the unmixed and mixed groups. Moreover, the international students in the mixed groups seemed to be more interactive than the Chinese students. These results suggest that in addition to the confidence that comes with language fluency, the international students’ attitudes toward the PBL courses may be a contributing factor. That is, the international students may have considered the interactions less of a serious task-oriented process than the Chinese students did and instead believed that the more interactions, the more they would learn. This could also be a result of the influence of intercultural sensitivity on student interactions. Chinese students, as members of a collectivist society, act modestly and cautiously in group interactions [56], and this is not conducive to developing the preferred patterns in group interactions. Another possibility is that the Chinese tutor did not differ the teaching strategies used in the unmixed and mixed groups, and thus targeted strategies to facilitate interaction between students from different cultural backgrounds were lacking.

The second research question was to determine whether the ethnic composition of student groups, intercultural sensitivity, and individual characteristics (age, gender, and ethnicity) affected the three types of group interaction in medical PBL. The results of the hierarchical regression analysis confirm that ethnic composition was a significant negative predictor of interactions involving exploratory questions and cumulative reasoning. Specifically, the ethnically mixed groups had fewer of these types of group interactions. This differs from previous studies that have reported positive collaborative learning experiences among ethnically diverse students [4, 6, 57]. According to schema theory [58], the greater the amount of cross-cultural contact in the classroom, the greater the student engagement. This study adds to the literature by examining the effect of intercultural contact in terms of the level of group interaction in a medical PBL setting. From this perspective, being able to give correct answers reflects a precise understanding of the topic, which means that simply placing members of multiple ethnic groups in a classroom for small group learning does not ensure profitable learning-oriented interactions. This finding agrees with previous studies that have reported negative collaborative learning experiences for ethnically heterogeneous groups of students [18, 59], as discussed, factors such as students’ intercultural sensitivity, level of English proficiency, attitudes toward the course, and cultural background potentially explain the significantly lower level of interaction by the Chinese students in the mixed groups. In the context of small group learning for medical students, greater multicultural exposure alone is insufficient. Students must be prepared to engage in effective cross-cultural interactions.

Interestingly, not only was intercultural sensitivity a positive predictor of two types of group interaction, it was also a stronger predictor than group ethnic composition. These findings reflect the Sociocultural Theory and echo the academic risk perspective in collaborative learning environment. Students perform better when they are prepared to deal with cultural differences in their learning environment. Although causal inference can’t be warranted by the design of this study, students with higher ISS level could better take full advantage of cultural diversity to complete PBL task, as they can better develop a positive emotion towards understanding and appreciating cultural differences and thus improves appropriate and effective behavior in intercultural interactions – a finding consistent with the previous research that reported a positive correlation between intercultural sensitivity and student engagement [31, 57]. This study’s unique contribution is the finding that intercultural sensitivity explains the difference between ethnically unmixed and mixed groups’ interactions involving exploratory questions and cumulative reasoning in a medical PBL setting. Students with high levels of intercultural sensitivity seem to function as active, skilled supporters in group interactions in both unmixed and mixed groups. Our results therefore help answer the question raised in previous studies of how to strategically design effective learning environments that encourage ethnically diverse students to interact and collaborate in beneficial ways [18, 60]. Therefore, one effective strategy would be to develop students’ cultural sensitivity before they engage in small group learning. Although the importance of intercultural competence is widely recognized by Chinese medical educators, to the best of our knowledge, formal intercultural competency training programs for undergraduate students are scarce or even absent in Chinese medical schools [61]. There is a strong need for policy makers and educators to develop a formal intercultural competency curriculum for undergraduates, including international students, as a preparatory course before they enter multicultural learning environments.

The results of this study indicate that there was no significant change in the intercultural sensitivity of either the Chinese or foreign students after they experienced ethnically mixed group learning. These findings provide some support for the view that simple multicultural contact does not enhance intercultural sensitivity in the context of medical PBL. Although Allport’s classic contact theory [40] suggests that the key to developing intercultural competence is having the opportunity to interact with people of different cultures, Allport also emphasized that under certain conditions (i.e., equal status, pursuit of common goals, and support from authorities), people from different ethnic and cultural backgrounds are more likely to respond favorably to one another as contact increases. This engagement further influences their attitudes and behaviors toward people of different ethnic and cultural backgrounds [62]. Hence, this study shows that rationally arranging multiethnic groups after considering their characteristics, rather than simply putting them together, may lead to profitable interactions and the development of intercultural sensitivity. PBL tutors should use thoughtful strategies to design effective group learning environments, such as managing interactions between students of different ethnicities, languages, and cultural competencies [63]. PBL tutors should also be trained in how to deal with ethnically diverse groups on a variety of issues.

The unmixed and mixed groups did not differ in their interactions related to handling conflicts, and such interactions are not predicted by a group’s ethnic composition or intercultural sensitivity. A reasonable explanation for this is that the students were not aware of conflict or did not experience it during the group interactions. Alternatively, as conflict involves uncertainty, it may have been perceived as a negative aspect of the group’s productivity and thus the students may have adopted an ambiguous attitude toward it [22]. However, this explanation does not seem to apply to the international students. The results show that the foreign students scored lower than the Chinese students in the group interactions that involved handling conflict. This phenomenon may need to be further explored from the cultural orientation perspective.

The roles of age, gender, and ethnicity in the three types of group interaction were also examined using hierarchical regression analysis. In line with general expectations, older group members performed better on the three types of group interaction, as they have more cognitive resources available because of their age. The groups’ ethnic heterogeneity was a significant predictor of their interactions in PBL. Specifically, the more foreign students there were in a group, the stronger the inhibitory effect on the Chinese students’ interactions. Gender was not a significant predictor, which contradicts previous studies that have suggested that gender heterogeneity in a group is negatively correlated with students’ interactions among early adolescents [42, 43]. Numerous studies have examined the effect of demographic diversity, such as age or gender, on group performance with inconsistent results [64–66]. This inconsistency may be related to the context dependence of demographic diversity. Specifically, contextual variables that make demographic features more or less prominent may affect the results. Some contextual variables such as majors, disciplines, and learning environments may decrease or increase the prominence of certain indicators of diversity.

## Limitations, practical implications, and recommendations for future research

A limitation of this study is that only the intercultural sensitivity of the students was analyzed rather than other variables at the individual level (i.e., overseas travel/study experience, self-perceived English proficiency, perceived stress, status, etc.) that may affect intercultural sensitivity [67, 68]. To provide additional reference information for designing multiethnic group learning curricula for medical students, future studies could examine other personal characteristics associated with intercultural sensitivity and evaluate their relationships to group interactions in multiethnic group learning environments.

This study examined the important role of intercultural sensitivity in multiethnic group learning, described issues encountered by students in medical PBL in China, and presented issues worthy of further research. The practical implications include the need to effectively increase students’ intercultural sensitivity before they engage in group learning and the need to develop new strategies that PBL tutors can use to encourage interaction in multiethnic groups. Examples of such strategies include defining and applying principles of rational subgrouping and being sensitive to the tensions that arise during interactions due to cultural differences and language barriers, and dealing with them appropriately. Future research is needed to further illuminate how universities can best meet the challenge of creating the optimal group learning environment for students of all ethnicities.

## Conclusion

This study extends the literature on medical students’ group interactions in PBL by exploring the role of group ethnic composition, intercultural sensitivity, and certain personal characteristics. The results indicate that for group interactions related to exploratory questions and cumulative reasoning, ethnic composition is a significant negative predictor and intercultural sensitivity is a strong positive predictor. In addition, group heterogeneity in terms of age and ethnicity is also a significant predictor of group interactions in medical PBL. These findings provide insight into how to strategically create effective learning environments in which multiethnic groups can interact and collaborate successfully.

## Data Availability

The dataset used during the study is available from the corresponding author on reasonable request.
